# Relationship between Malaria and ABO Blood Types in East China

**DOI:** 10.1155/2017/8163762

**Published:** 2017-11-07

**Authors:** Xuan Zhang, Meifang Yang, Hong Zhao, Jianhua Hu, Lanjuan Li

**Affiliations:** State Key Laboratory for Diagnosis and Treatment of Infectious Diseases, Collaborative Innovation Center for Diagnosis and Treatment of Infectious Diseases, The First Affiliated Hospital, College of Medicine, Zhejiang University, 79 Qingchun Road, Hangzhou 310003, China

## Abstract

**Objectives:**

This study aims at investigating the relationship between malaria and blood group types in east China.

**Methods:**

Between 1 January 2011 and 31 March 2017, 99 malaria patients were enrolled for the study. Laboratory tests were conducted on their infection status and blood types. Clinical data of the participants were retrieved for analysis.

**Results:**

There was no mortality during the period of study. Overall, 90 (90.91%) of the patients were positive for* Plasmodium falciparum*, 8 (8.08%) were infected with* Plasmodium vivax*, and only 1 (1.01%) was infected with* Plasmodium malariae*. The most common blood group among the participants was group O (38.38%) followed by blood groups A, B, and AB, with 32.32%, 22.22%, and 7.07% cases, respectively. There was no significant relationship between the prevalence of malaria and ABO blood types (*P* > 0.05). In the blood group O, the prevalence of haemolytic-uremic syndrome and cerebral malaria was 13.16% and 5.25%, respectively, which was lower than that of the other three blood types (*P* > 0.05).

**Conclusion:**

There was no mortality among the malaria patients in this study. The blood group O was the most common blood type. Due to small sample size of data, there was no significant association between ABO blood types and malaria infection.

## 1. Introduction

Malaria is a life-threatening parasitic disease transmitted by mosquitoes. Positive prevention and treatment for malaria have resulted in a wide-scale reduction in incidence and mortality; however, 198 million cases (uncertainty range: 124–283 million) and 584,000 deaths (range: 367,000–755,000) still occur yearly [1].

The relationship between ABO blood types and malaria susceptibility has been studied by several researchers; however, the results have been contradictory. Bayoumi et al. [2] observed no association between malaria prevalence and ABO blood types in central Sudan. Similarly, a study in Nigeria reported no significant association between ABO blood types and malaria infection [3]. However, other studies indicated a significant association between malaria and ABO blood types. Bedu-Addo et al. showed a clear protective effect of blood group O against malaria in primipara [4]. Research in Gabon reported a significant association between blood group A and severe malaria [5].

Among the global malaria burden, 90% has been estimated to occur in Sub-Saharan Africa [6]; hence, research on the association between ABO blood types and malaria has been concentrated in Africa. Studies on the association between ABO blood types and malaria among Chinese adults are extremely rare. However, malaria is one of the major parasitic diseases with a wide distribution in China [7]. Therefore, this study aims to determine the relationship between ABO blood types and malaria among adults in east China.

## 2. Methods

### 2.1. Study Design

This retrospective study was conducted in the State Key Laboratory for Diagnosis and Treatment of Infectious Diseases, Department of Infectious Diseases, the First Affiliated Hospital, College of Medicine, Zhejiang University, China. The research was approved by the Ethics Committee of the First Affiliated Hospital of Zhejiang University, which waived the need for consent because the study was retrospective and the data were analysed anonymously.

### 2.2. Study Population

Between 1 January 2011 and 31 March 2017, malaria patients aged over 18 years were admitted into the First Affiliated Hospital, College of Medicine, Zhejiang University, China. All patients were hospitalized due to fever. Patients with positive bacterial blood cultures or those with obvious bacterial, viral, or other parasitic infections were excluded. For the control group, we randomly selected 100 subjects from those that presented with fever but were negative for malaria. Of the control group, patients with complicated underlying diseases were excluded. The demographic and clinical details of the malaria patients were obtained from the case records of the patients.

### 2.3. Methods

Two reviewers at a reference parasitology laboratory were blinded of the thin and thick blood smears and independently confirmed the malaria diagnoses. The blood groups were determined using the forward and reverse method. Haematological parameters, which included haemoglobin, total leukocyte count, and platelet count of each patient, were done on an automated cell counter.

### 2.4. Statistical Analysis

The statistical analysis was performed using SPSS version 18.0 (SPSS, Chicago, IL, USA). Chi-square test was conducted to calculate and compare the categorical variables. The measurement data between different ABO blood types were compared with single-factor analysis of variance. *P* value < 0.05 was considered statistically significant for all analyses.

## 3. Results

### 3.1. Patients

In this study, 99 malaria patients (41.82 ± 14.68 years) were enrolled, with a male preponderance (90.91%). All the 99 malaria patients were treated and discharged from the hospital or had improved and were discharged with drugs. No mortality was recorded during the period of study. One hundred patients (41.77 ± 14.53 years old, 60 males) who were hospitalized due to fever without malaria were randomly selected as control group.

Of the 99 malaria patients, 90 (90.91%) were infected with* Plasmodium falciparum*, 8 (8.08%) were infected with* Plasmodium vivax,* and only 1 (1.01%) was infected with* Plasmodium malariae*. Moreover, 97 (97.98%) patients were imported malaria cases. Before being diagnosed with malaria, 94 patients worked or travelled in Africa and 3 patients travelled in Southeast Asia.

During treatment, 75 patients received artemisinin and 24 patients received dihydroartemisinin piperaquine phosphate tablets. Pyronaridine phosphate, chloroquine, and primaquine were used in 6, 4, and 11 patients, respectively.

### 3.2. ABO Blood Group

The most common blood group among the 99 malaria patients was group O (38.38%) followed by blood groups A (32.32%), B (22.22%), and AB (7.07%) respectively. The distribution of ABO blood types among the control group was A (32%), B (23%), O (32%), and AB (13%) respectively. There were no differences in the distribution of ABO blood group types among the malaria patients and control group (Table 1). Moreover, laboratory data, which included leukocyte counts, haemoglobin, platelets, and C-reactive protein, had no statically significant differences in malaria patients and ABO blood types (Table 2). Among the malaria patients who had haemolytic-uremic syndrome and cerebral malaria, the blood type O had the lowest prevalence. However, no statistically significant relationship was found between individual complications and ABO blood types (Table 3).

## 4. Discussion

The relationship between ABO blood types and malaria was first suggested over 50 years ago [8], and new information have since emerged, with the prevailing controversies. In this study, 97.98% of the malaria infections were imported malaria cases. The main Chinese populations working in Africa are males. Hence, this finding can explain why malaria patients had a male preponderance (90.91%). This finding agrees with previous studies [9, 10]. This study was aimed at investigating the role of ABO blood types on malaria risk among the Chinese people. However, no statistically significant relation was found between malaria prevalence and ABO blood types, which was consistent with previous studies [2, 3, 11].

Among the malaria patients, blood group O had the highest prevalence with 38.38%. This result agrees with previous studies, which reported a high frequency of blood group O in tropical regions with rampant malaria [3, 9, 11, 12]. However, contrary to our observation, Deepa et al. [13] stated blood group B as the dominant blood type in their study. We speculated that the difference was induced by the different distributions of ABO blood types in various races.

Among malaria patients with blood group O, the prevalence of haemolytic-uremic syndrome and cerebral malaria was 13.16% and 5.25%, respectively, which was lower than that of the other three blood types. Therefore, we considered blood group O to be prone to less severe malaria, although no statistically significant relation was found. Toure et al. [14] and Hegde et al. [11] found the same phenomenon. Compared with other blood groups, blood group O is less prone to rosetting, which results in reduced complications in malaria patients [11]. Rosetting is a highly established virulence factor of* Plasmodium falciparum* infection [11, 12, 15].

Our study has some limitations. First, the small numbers of malaria patients and control group may lead to statistical errors. Second, malaria patients in this study were concentrated in the Zhejiang Province; the data may be insufficient to evaluate the relationship between ABO blood types and malaria in east China. Future studies with an increased scale are warranted in more regions to explore whether the result is similar in other populations.

## 5. Conclusions

In conclusion, there was no mortality among the malaria patients in this study. There was no significant association between ABO blood types and malaria infection. However, the blood group O is the most common blood type in malaria patients in east China, who are prone to less severe malaria. Therefore, malaria patients with blood group O seemingly have mild clinical outcomes.

## Figures and Tables

**Table 1:** The distribution of ABO blood types in malaria patients and the control group (febrile patients without malaria).

ABO blood types	Malaria (*n* = 99)	Controls (*n* = 100)	*P* value
A	32	32	0.96
B	22	23	0.90
O	38	32	0.35
AB	7	13	0.16

**Table 2:** Laboratory data of different ABO blood types in malaria patients.

Laboratory data		ABO blood types				*P* value
		A	B	O	AB	
Leukocyte counts (×10^9^/L)	5.63 ± 2.27	5.46 ± 2.05	4.82 ± 1.88	6.17 ± 2.69	6.03 ± 1.08	0.15
Haemoglobin (g/L)	102.29 ± 31.91	101.44 ± 29.20	106.95 ± 29.44	99.84 ± 34.59	104.86 ± 41.08	0.86
Platelets (×10^9^/L)	71.54 ± 45.07	73.53 ± 50.67	62.64 ± 34.96	69.95 ± 41.29	99.00 ± 62.62	0.49
C-reactive protein (mg/L)	78.27 ± 47.28	66.40 ± 48.25	91.29 ± 41.62	77.97 ± 47.39	89.89 ± 55.66	0.28

**Table 3:** The distribution of ABO blood types in malaria patients with haemolytic-uremic syndrome and cerebral malaria.

Blood group	Malaria (*n* = 99)	Complication			
		Haemolytic-uremic syndrome (*n* = 14)	*P* value	Cerebral malaria (*n* = 10)	*P* value
A	32	5 (15.63%)	0.770	4 (12.5%)	0.85
B	22	3 (13.64%)	0.939	3 (13.64%)	0.89
O	38	5 (13.16%)	0.825	2 (5.25%)	0.36
AB	7	1 (14.29%)	0.991	1 (14.29%)	0.54
